# Does Dietary Deoxynivalenol Modulate the Acute Phase Reaction in Endotoxaemic Pigs?—Lessons from Clinical Signs, White Blood Cell Counts, and TNF-Alpha

**DOI:** 10.3390/toxins8010003

**Published:** 2015-12-23

**Authors:** Tanja Tesch, Erik Bannert, Jeannette Kluess, Jana Frahm, Susanne Kersten, Gerhard Breves, Lydia Renner, Stefan Kahlert, Hermann-Josef Rothkötter, Sven Dänicke

**Affiliations:** 1 Institute of Animal Nutrition, Friedrich-Loeffler Institute (FLI), Federal Research Institute of Animal Health, Bundesallee 50, 38116 Braunschweig, Germany; tanja.tesch@fli.bund.de (T.T.); erik.bannert@fli.bund.de (E.B.); jana.frahm@fli.bund.de (J.F.); Susanne.kersten@fli.bund.de (S.K.); sven.daenicke@fli.bund.de (S.D.); 2 Institute for Physiology, University of Veterinary Medicine, Foundation, Bischofsholer Damm 15, 30173 Hannover, Germany; gerhard.breves@tiho-hannover.de; 3 Institute of Anatomy, Otto von Guericke University Magdeburg, Leipziger Str. 44, 39120 Magdeburg, Germany; lydia.renner@med.ovgu.de (L.R.); stefan.kahlert@med.ovgu.de (S.K.); hermann-josef.rothkoetter@med.ovgu.de (H.-J.R.)

**Keywords:** pig, deoxynivalenol, lipopolysaccharide, liver, leukocytes, clinical symptoms, tumor necrosis factor alpha, body core temperature

## Abstract

We studied the interaction between deoxynivalenol (DON)-feeding and a subsequent pre- and post-hepatic immune stimulus with the hypothesis that the liver differently mediates the acute phase reaction (APR) in pigs. Barrows (*n* = 44) were divided into a DON-(4.59 mg DON/kg feed) and a control-diet group, surgically equipped with permanent catheters pre- (*V. portae hepatis*) and post-hepatic (*V. jugularis interna*) and infused either with 0.9% NaCl or LPS (7.5 µg/kg BW). Thus, combination of diet (CON *vs.* DON) and infusion (CON *vs.* LPS, jugular *vs.* portal) created six groups: CON_CON_jug._-CON_por._, CON_CON_jug._-LPS_por._, CON_LPS_jug._-CON_por._, DON_CON_jug._-CON_por._, DON_CON_jug._-LPS_por._, DON_LPS_jug._-CON_por._. Blood samples were taken at −30, 15, 30, 45, 60, 75, 90, 120, 150, 180 min relative to infusion and analyzed for leukocytes and TNF-alpha. Concurrently, clinical signs were scored and body temperature measured during the same period. LPS as such induced a dramatic rise in TNF-alpha (*p* < 0.001), hyperthermia (*p* < 0.01), and severe leukopenia (*p* < 0.001). In CON-fed pigs, an earlier return to physiological base levels was observed for the clinical complex, starting at 120 min *post infusionem* (*p* < 0.05) and persisting until 180 min. DON_LPS_jug._-CON_por._ resulted in a lower temperature rise (*p* = 0.08) compared to CON_LPS_jug._-CON_por._. In conclusion, APR resulting from a post-hepatic immune stimulus was altered by chronic DON-feeding.

## 1. Introduction

The B-trichothecene deoxynivalenol (DON) is a mycotoxin mainly produced by the plant pathogens *Fusarium graminearum* and *F. culmorum*. It is often detected in cereal grains, especially wheat and maize. These are major portions of farm animal’s diets, which are therefore frequently contaminated with toxicologically relevant levels. Pigs are known as the most DON-sensitive species [1,2] and symptoms such as vomiting, inappetence, and reduced weight gain are often related to DON-contamination of the pig feed. Moreover, DON effects are dependent on dosing regime and frequency of exposure and can be immunosuppressive at acute high-doses, e.g., reflected by rapid increase of leukocyte apoptosis or immunostimulatory at low-doses, e.g., resulting in an increased expression of cytokines. It is conceivable that a large proportion of animals in swine production might encounter chronic dietary DON exposure during their lifetime and, given the infectious pressure in the production site, might also be co-exposed to a systemic infection such as Salmonellosis, Campylobacter, or *E. coli* infections [3,4]. Because DON has been reported to modify the organism’s immune response, this poses the question whether pigs pre-exposed to dietary mycotoxins react with an altered response, e.g., in the clinical progression of an occurring systemic infection and thus the ability of the organism to deal with this disease. Investigating such a potential interaction of mycotoxin exposure and systemic infections one needs to use an established infection or inflammation animal model enabling the researcher to discern the known effects of the infection from the to-be-investigated additional mycotoxin impact. The endotoxin (LPS) is a well-established model substance, challenging the immune system and thus simulating an inflammatory state usually present in a systemic infection. LPS is a component of the outer cell membrane of gram-negative bacteria and consists of a hydrophobic domain as the source of toxicity (lipid A), a core region (oligosaccharides), and a hydrophilic O-specific chain responsible for most antigenic properties (polysaccharide) [5].

Data from rodent studies reported synergistic effects between DON and LPS with respect to the acute phase reaction (APR) in particular the induction of pro-inflammatory cytokine expression [6,7]. Furthermore, LPS priming potentiated not only the pro-inflammatory response to DON exposure but also the toxicity of both [8,9].

In the challenge model, LPS has a high stimulatory potential for the innate and acquired immune system due to the interaction with different types of leukocytes initiating, amongst other things, the biosynthesis of various mediators of inflammation, such as TNF-alpha. This is the first pro-inflammatory cytokine in the cytokine cascade playing a major role in fever induction as well as in the occurrence of related clinical signs as the first and easiest observable changes in vivo. Thus, the response of the immune system to LPS in animals pre-exposed to DON might be reflected in altered differential blood cell counts and clinical manifestation of an APR resulting from increased levels of TNF-alpha and other inflammatory mediators [5].

Both DON, as a feed contaminant, and LPS, as part of commensal bacteria, mainly enter the organism via the gastrointestinal tract (GIT) where they can be absorbed into the blood stream [1,10,11,12] The GIT blood flow is drained into the portal vein (portal drained viscera, PDV) and all absorbed substances such as nutrients or even toxins enter the liver via this route. The liver is not only the central metabolic organ for the key nutrients such as carbohydrates and proteins, but also processes and eliminates toxic agents, e.g., DON is glucuronidated in the liver [13]. Furthermore, systemic inflammatory reactions are also facilitated via hepatic Kupffer cells contributing to local and systemic effects. Therefore, substances absorbed from the GIT and entering the liver in a so-called first pass are likely to elicit a different response compared to their counterparts in systemic circulation, gaining entry to the liver only in a second pass. Although this is a crucial point, there are only scarce data discerning immune stimulation and its response pre- and post-hepatically, in particular regarding myco- and endotoxin exposure. Thus, we wanted to test the hypothesis that the liver differently mediates the systemic inflammatory response to an acute LPS-stimulus in chronically DON-fed pigs. Therefore, we employed a pig model enabling portal (pre-hepatic) and peripheral (post-hepatic) access for assessing the liver’s contribution to the pig’s response to myco- and endotoxin exposure.

## 2. Results

### 2.1. Clinical Score

**Figure 1:**
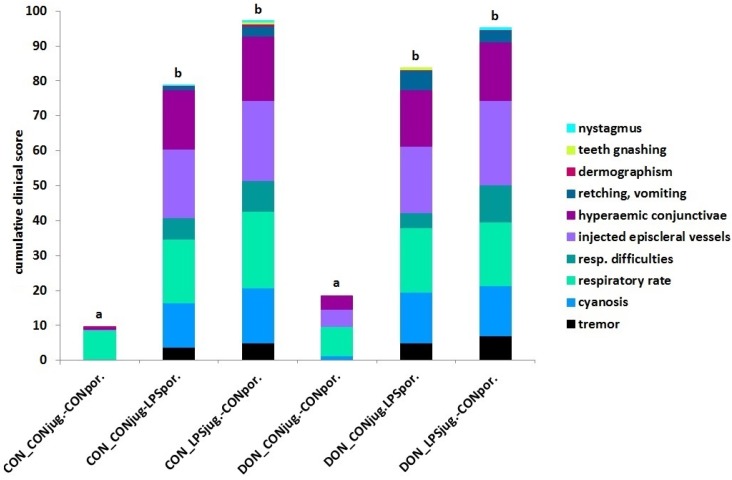
The cumulative clinical score (CCS) represents the entire clinical complex induced by the six experimental treatments, *i.e.*, chronic feeding strategy (CON *vs.* DON) and acute challenge situation (CON *vs.* LPS). Each CCS bar consists of maximum 10 clinical symptoms scored at 13 points in time over the entire observation period of 210 min for each group. LPS-infused groups had a significant higher CCS and considerably more symptoms than control-infused groups, irrespective of dietary treatment. Data represent LSmeans (PSEM ± 8.6) and statistical main effects were distributed as follows: *p_group_* < 0.001. Bars with no common superscripts (a,b) are significantly different (*p* < 0.001).

Ten clinical symptoms characterizing an inflammatory reaction were observed in a time kinetic manner and scored for their presence and severity for all experimental groups. Data for all symptoms and all times tested were subsumed in a cumulative clinical score (CCS) for each experimental group representing a clinical complex. All LPS exposed pigs had a significantly higher CCS compared to control-infused groups. Viewing the four LPS-exposed group revealed that portal-infused groups showed numerically lower CCS compared to their jugular-infused counterparts, but this could not be statistically verified. DON-feeding alone (DON_CON_jug._-CON_por._) did not alter clinical symptoms and combined with LPS did not alter the LPS-reaction. At least 5 out of 10 detected individual clinical symptoms indicative for an acute endotoxaemia were represented in LPS-infused groups, while both control infusion groups displayed only sporadically adverse symptoms (Figure 1).

**Figure 2:**
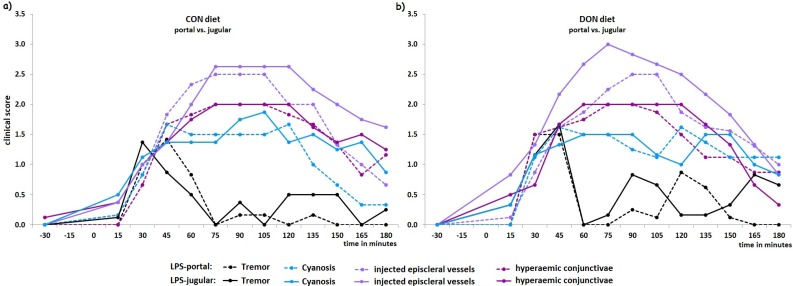
LPS caused a sequential series of four key symptoms and the kinetic of those individual clinical signs is depicted in the two upper graphs for each dietary treatment. (**a**) showing CON-fed and (**b**) DON-fed groups. Tremor reached its highest level already between 30 and 45 min while the other symptoms showed there maximum degree between 75 and 105 min. Thereafter, from 120 min onwards, symptoms declined slowly to the base level. Data represent LSmeans (PSEM_Tremor_ ± 0.07, PSEM_injected episcleral vessels/cyanosis_ ± 0.15, PSEM_hyperaemic conjunctivae_ ± 0.11) and statistical main effects were distributed as follows: cyanosis *p_group_* < 0.001, *p_time_* < 0.001, *p_group × time_* < 0.01, tremor/hyperaemic conjunctivae/injected episcleral vessels *p_group_* < 0.001, *p_time_* < 0.001, *p_group × time_* < 0.001. The cumulative clinical score (CCS) for each point in time is presented in (**c**) and the statistical difference (*p*-value) between jugular and portal infusion within each feeding group is provided. Data represent LSmeans and statistical main effects were distributed as follows: *p_group_* < 0.001, *p_time_* < 0.001, *p_group × time_* < 0.001.

The most pronounced symptoms observed in all LPS-pigs were tremor, cyanosis, injected episcleral vessels and hyperaemic conjunctivae showing a coherent time kinetic (Figure 2a,b), whereas nystagmus, teeth gnashing, and dermographism were sporadic symptoms that occurred only in a few LPS-infused animals (*n* = 5 out of 28 LPS-pigs). Although labored respiration, increased respiratory rate, retching, and vomiting were also mostly detected in LPS-infused pigs, these symptoms did not show a clear sequence of time. The four key symptoms (Figure 2a,b) had a uniform sequence starting between 15 and 30 min *post infusionem* (*p.i.*) with tremor and cyanosis followed by injected episcleral vessels and hyperemic conjunctivae in LPS-groups. Control-infused groups showed no adverse clinical symptoms and thus were not included in Figure 2a,b. We calculated also the CCS for each point in time (identical to Figure 1) in order to discriminate the influence of DON and LPS on the course of the clinical symptoms (Figure 2c). Here we observed that the site of LPS-infusion, *i.e.*, pre- or post-hepatically, had a stronger impact on the clinical progression as compared to the mycotoxin influence. Within CON-fed groups portal infusion caused a significantly earlier return (120 min *p.i.*, *p* = 0.05) to physiological levels compared to jugular infusion and this effect persisted until 180 min. In DON-fed pigs, jugular infusion showed a significantly higher score in clinical symptoms at 15 min *p.i.* (*p* = 0.04) compared to their portal infused counterparts, but no other differences could be verified. 

**Table:** 

**c)**	**Time in Minutes**												
	−30	15	30	45	60	75	90	105	120	135	150	165	180
CON_CON_jug._-LPS_por._	0.7	1.9	5.7	8.1	7.9	7.7	8.3	7.6	7.1	6.0	5.4	4.9	3.7
CON_LPS_jug._-CON_por._	1.4	2.4	7.3	8.4	8.6	8.1	8.6	9.8	9.8	8.6	8.3	7.8	8.6
*p*-values	0.62	0.69	0.25	0.82	0.56	0.76	0.80	0.10	0.05	0.05	0.04	0.03	<0.001
DON_CON_jug._-LPS_por._	0.9	1.7	8.4	10.1	8.6	7.9	7.4	7.0	9.1	7.3	6.8	6.2	7.3
DON_LPS_jug._-CON_por._	1.0	4.7	8.0	9.5	9.5	8.8	9.3	8.8	8.3	8.5	7.2	7.0	4.7
*p*-values	0.92	0.04	0.77	0.65	0.50	0.50	0.18	0.20	0.58	0.40	0.79	0.59	0.07

### 2.2. Body Temperature

**Figure 3:**
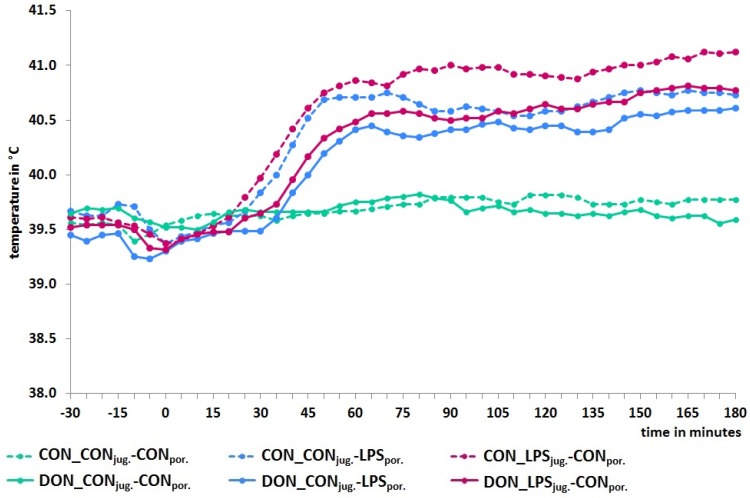
Body core temperature measurement (in 5 min intervals) for all experimental groups. All LPS-infused groups increased significantly and showed a hyperthermia in contrast to control-infused groups. DON-feeding had also a marked effect in LPS-infused animals and showed consistently lower temperature (~0.5 °C) as compared to their control-fed counterparts. Data represent LSmeans (PSEM ± 0.02) and statistical main effects were distributed as follows: *p_group_* < 0.01, *p_time_* < 0.001, *p_group × time_* < 0.001.

Body temperature was measured every 5 min with an intra-abdominal temperature logger (body core temperature). Compared to control-infused animals showing no increase in temperature during the entire observation period, LPS-infusion induced a significant hyperthermia with an increase of ~1.5 °C, starting from 30 min *p.i.*, irrespective of infusion site or diet. Additionally, a marked diet effect was found in jugular-infused LPS-pigs: DON-fed animals (*p* = 0.08) exhibited consistently lower temperature (~0.5 °C) as compared to their LPS-infused, CON-fed counterparts (Figure 3). When comparing the two feeding-groups at each point in time, *t*-test revealed that, during the period 30 to 115 min *p.i.*, DON-fed pigs displayed significantly lower temperatures (*p* < 0.05).

### 2.3. White Blood Cell Counts

White blood cell counts were analyzed in arterial and pre- and post-hepatic venous blood samples, whereby no statistical differences were found between sampling sites (Table 1). At all sampling locations a severe leukopenia (*p* < 0.001) was detected in response to LPS-infusion, with counts falling to a minimum of 2.2 × 10^3^/µL, irrespective of infusion site and diet, starting at 30 min *p.i*.. However, group DON_LPS_jug._-CON_por._ exhibited an even earlier leukopenia, already significantly present at 15 min compared to −30 min (*p* < 0.05), whereas the other LPS-infused groups showed a significant decrease at 30 min compared to −30 min (*p* < 0.05). Within the study period no return of leukocyte counts to base levels was observed in LPS-groups.

Prior to the infusion regime at −30 min, DON-fed pigs showed significantly (*p* = 0.04) higher leukocyte counts than CON-feed animals (16.7 *vs.* 14.6 × 10^3^/µL). This dietary effect was detectable for control-infused groups throughout the study period, with leukocyte counts in DON-fed animals showing consistently higher values (~2 × 10^3^/µL) as compared to CON-group.

**Table 1:** Time kinetics of total leukocyte counts (10^3^/µL). Physiological range 8–16 × 10^3^/µL [14].

Experimental Groups		Time in Minutes										*n*
		−30	15	30	45	60	75	90	120	150	180	
**carotid artery**	CON_CON_jug._-CON_por._	13.53 ^a^	13.27 ^a,b^	13.33 ^a^	13.71 ^a^	13.47 ^a^	13.19 ^a^	13.54 ^a^	13.57 ^a^	13.78 ^a^	14.34 ^a^	7
	CON_CON_jug._-LPS_por._	14.07 ^a^	10.47 ^a,b^	6.80 ^b^	4.52 ^b^	4.38 ^b^	3.19 ^b^	2.57 ^b^	2.21 ^b^	2.28 ^b^	2.75 ^b^	4
	CON_LPS_jug._-CON_por._	15.55 ^a^	10.61 ^a,b^	7.39 ^b^	4.15 ^b^	4.36 ^b^	3.66 ^b^	3.11 ^b^	2.33 ^b^	2.68 ^b^	3.19 ^b^	7
	DON_CON_jug._-CON_por._	16.34 ^a^	15.83 ^a^	15.88 ^a^	16.37 ^a^	15.85 ^a^	16.62 ^a^	16.17 ^a^	16.58 ^a^	16.85 ^a^	17.35 ^a^	6
	DON_CON_jug._-LPS_por._	17.39 ^a^	11.73 ^a,b^	6.49 ^b^	4.76 ^b^	5.49 ^b^	4.49 ^b^	3.63 ^b^	3.23 ^b^	3.33 ^b^	3.93 ^b^	5
	DON_LPS_jug._-CON_por._	14.80 ^a^	9.36 ^b^	4.81 ^b^	3.47 ^b^	3.98 ^b^	3.16 ^b^	2.72 ^b^	2.24 ^b^	2.25 ^b^	2.36 ^b^	5
**jugular vein**	CON_CON_jug._-CON_por._	13.97 ^a^	13.6 ^a,b^	13.74 ^a^	13.53 ^a^	13.46 ^a^	13.61 ^a^	14.09 ^a^	14.13 ^a^	14.25 ^a^	14.92 ^a^	8
	CON_CON_jug._-LPS_por._	14.15 ^a^	10.88 ^a,b^	6.68 ^b^	4.62 ^b^	4.78 ^b^	3.30 ^b^	2.85 ^b^	2.26 ^b^	2.48 ^b^	2.88 ^b^	6
	CON_LPS_jug._-CON_por._	15.87 ^a^	11.39 ^a,b^	8.3 ^b^	4.37 ^b^	5.63 ^b^	3.87 ^b^	3.30 ^b^	2.76 ^b^	2.88 ^b^	3.36 ^b^	9
	DON_CON_jug._-CON_por._	16.39 ^a^	16.11 ^a^	16.36 ^a^	16.31 ^a^	15.87 ^a^	16.15 ^a^	15.94 ^a^	16.92 ^a^	16.97 ^a^	17.07 ^a^	7
	DON_CON_jug._-LPS_por._	18.37 ^a^	12.74 ^a,b^	7.19 ^b^	5.80 ^b^	6.24 ^b^	4.31 ^b^	3.35 ^b^	2.74 ^b^	3.26 ^b^	3.58 ^b^	7
	DON_LPS_jug._-CON_por._	14.90 ^a^	9.96 ^b^	5.88 ^b^	4.04 ^b^	4.42 ^b^	3.40 ^b^	2.82 ^b^	2.26 ^b^	2.34 ^b^	2.4 ^b^	5
**portal vein**	CON_CON_jug._-CON_por._	13.53 ^a^	13.77 ^a,b^	13.48 ^a^	13.63 ^a^	13.41 ^a^	13.91 ^a^	13.98 ^a^	13.38 ^a^	14.2 ^a^	14.16 ^a^	8
	CON_CON_jug._-LPS_por._	15.21 ^a^	10.35 ^a,b^	6.25 ^b^	4.43 ^b^	4.23 ^b^	3.31 ^b^	2.92 ^b^	2.43 ^b^	2.60 ^b^	2.9 ^b^	6
	CON_LPS_jug._-CON_por._	15.76 ^a^	11.36 ^a,b^	7.39 ^b^	3.77 ^b^	4.44 ^b^	3.77 ^b^	3.11 ^b^	2.64 ^b^	3.15 ^b^	3.4 ^b^	9
	DON_CON_jug._-CON_por._	16.49 ^a^	15.91 ^a^	16.43 ^a^	16.1 ^a^	16.27 ^a^	16.2 ^a^	16.07 ^a^	16.87 ^a^	16.5 ^a^	17.07 ^a^	7
	DON_CON_jug._-LPS_por._	18.06 ^a^	12.99 ^a,b^	7.39 ^b^	4.56 ^b^	5.54 ^b^	4.36 ^b^	3.37 ^b^	2.89 ^b^	3.00 ^b^	3.27 ^b^	7
	DON_LPS_jug._-CON_por._	15.02 ^a^	9.53 ^b^	6.62 ^b^	3.50 ^b^	3.68 ^b^	3.16 ^b^	2.74 ^b^	2.32 ^b^	2.26 ^b^	2.66 ^b^	5

Data represent LSmeans (PSEM ± 0.7) and statistical main effects were distributed as followed: *p_group_* < 0.001, *p_sampling site_* = 0.02, *p_time_* < 0.001, *p_group × sampling site × time_* < 0.001. Values with no common superscripts (a,b) are significantly different within columns (*p* < 0.05).

The decrease of total leukocytes was confirmed in white blood cell differentiation with a severe neutropenia and lymphopenia in LPS-infused groups while leukocyte types in both control groups were stable in the physiological range.

**Figure 4:**
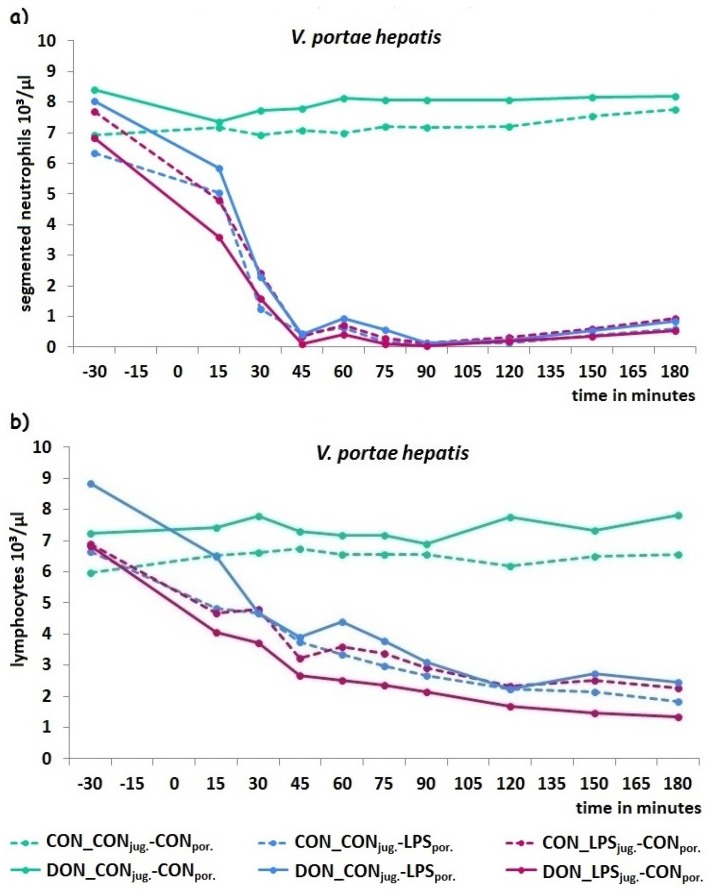
Kinetics of segmented neutrophil (**a**) and lymphocyte (**b**) counts in all experimental groups, with portal data exemplary for all sampling sites. LPS caused a severe neutrophil decrease starting at 15 min *p.i.*. Data represent LSmeans (SEM ± 0.3) and statistical main effects were distributed as followed: *p_group_* < 0.001, *p_sampling site_* = 0.03, *p_time_* < 0.001, *p_group ×sampling site × time_* < 0.001. LPS caused a more slowly decrease in lymphocytes, also starting at 15 min *p.i.*. Data represent LSmeans (SEM ± 0.3) and statistical main effects were distributed as followed: *p_group_* < 0.001, *p_sampling site_* < 0.001, *p_time_* < 0.001, *p_group × sampling site × time_* < 0.001.

Segmented neutrophils (×10^3^/µL or %) decreased in LPS infused groups, starting 15 min *p.i.* and falling abruptly below physiological range (4–9.6 × 10^3^/µL or 50%–60%; [14]) at 45 min, irrespective of infusion site or diet (Figure 4). In comparison, lymphocyte percentage increased significantly at 30 min *p.i.* while total counts decreased de facto due to the severe leukopenia addressed above (Table 1) gradually below the physiological values (2.8–8 × 10^3^/µL or 35%–50%; [14]). Monocytes (×10^3^/µL) and eosinophils (×10^3^/µL) decreased in LPS-infused groups significantly starting at 45 min *p.i.* while basophil total counts showed only slight variations, contributing to significant group, time and group × sampling site × time interaction. No change at all was found in banded neutrophils at all, representing immature neutrophils usually invading from bone marrow to replace the mature segmented neutrophils in blood.

### 2.4. TNF-Alpha

TNF-alpha, an early pro-inflammatory cytokine, was anaylyzed in pre- and post-hepatic venous blood samples, whereby no differences were found between blood sampling sites. Control-infused animals showed values <1 ng/mL during the entire experimental period, comparable to the respective base-level at −30 min.

**Figure 5:**
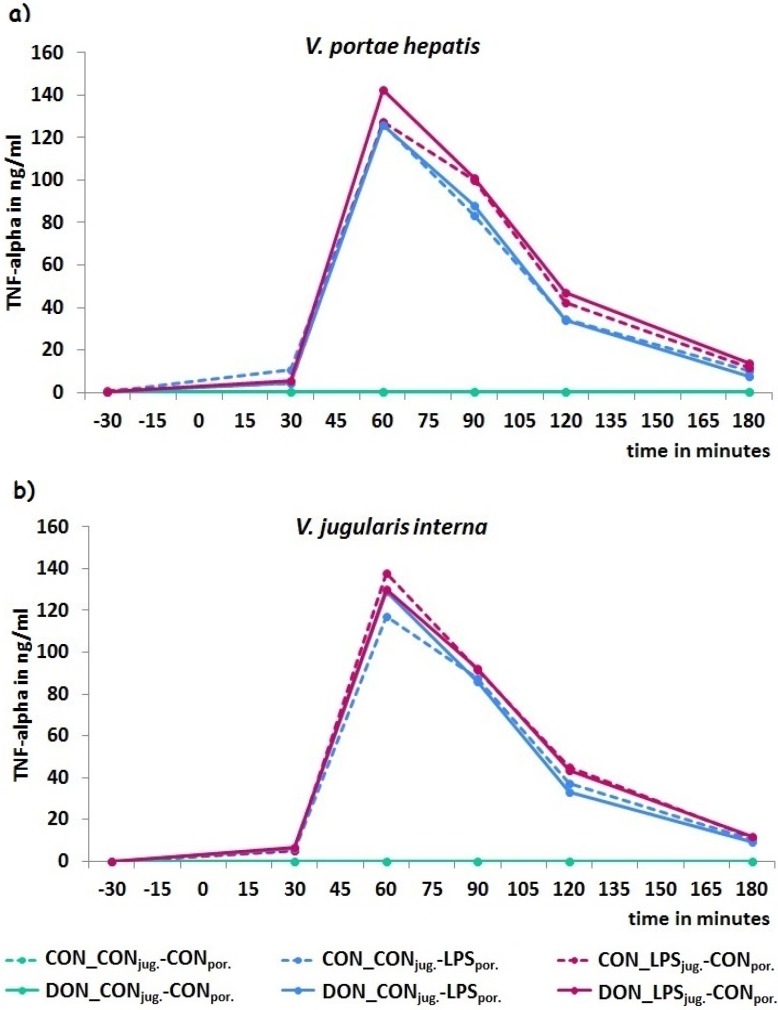
Kinetics of TNF-alpha measured in (**a**) portal and (**b**) jugular blood samples. Control-infused groups showed consistently unchanged TNF-alpha values of 0.4 ng/mL ± 0.03 during the entire experimental period. LPS-infusion elicited a strong increase of the cytokine with peak-values at 60 min, irrespective of dietary treatment or site of infusion. Data represent LSmeans (PSEM±) and statistical main effects were distributed as followed: *p_group_* < 0.001, *p_sampling site_* = 0.46, *p_time_* < 0.001, *p_group × sampling site × time_* < 0.001.

In LPS-infused groups a severe, sudden increase of TNF-alpha started at 30 min *p.i.* and peaked at 60 min (~140 ng/mL), irrespective of infusion site and diet. Subsequently, TNF-alpha decreased to values below half the peak value until 120 min and returned almost back to baseline at 180 min *p.i.* (Figure 5).

## 3. Discussion

Earlier experiments investigating the interactions between oral DON exposure and intravenous LPS administration in pigs were limited to examinations at the systemic level only and thus neglected the crucial role of the liver in the clearance of absorbed DON and LPS [15]. Therefore, we specifically addressed the role of the liver by infusing LPS into the portal vein, mimicking a gastro-intestinal originated flooding of the liver by LPS. This increased hepatic LPS-influx has been proposed to be mediated by a DON-induced impairment of the intestinal barrier [16,17,18]. Enabling LPS administration via the portal or systemic route in pigs exposed orally to either DON or CON diet, we wanted to test the hypothesis that the liver differently mediates the systemic inflammatory response as manifested by clinical signs as well as alterations in the plasma concentration of pro-inflammatory cytokines and white blood cell counts.

In our pigs we found typical signs for a systemic inflammatory response characterized by an increase in respiratory rate, reddened conjunctivae, injected episcleral vessels, shivering, retching and vomiting, as well as a marked fever response associated with a severe leukopenia based on neutropenia and lymphopenia, in all LPS-infused animals, without regard to any impact by DON-feeding or infusion site. Most of these symptoms we detected had a time-dependent uniform sequence starting 15 min *p.i*. and reaching their highest scores from 75 min *p.i.* onwards. Similar LPS-induced alterations were also found in previous studies with endotoxemic pigs in a comparable experimental setup [19] and in other large animal and rodent septic models [20,21].

The time-dependent sequence of symptoms is presumably depending on the sequential appearance of pro-inflammatory cytokines, released from immunocompetent cells [22,23]. In our LPS-infused pigs TNF-alpha reached its peak concentration at 1 h *p.i.*, which was associated with a simultaneous fever response as one of the main symptoms of a systemic inflammatory response. Data from studies with a similar experimental setup detected analogical clinical signs and these symptoms coincided with a rise in systemic plasma concentration of pro-inflammatory mediators (TNF-alpha, IL-6), clearly induced by LPS [15,23]. Based on the observed alterations, mentioned above, as well as the two different sites of infusion, we considered our endotoxemic porcine model as well-suited for investigating the systemic and hepatic effects of a LPS-induced APR. However, in the present study we failed to detect any differences in TNF-alpha kinetics caused by the pre- or post-hepatic entry of LPS into the liver. This result suggests that the amount of LPS entering the organism at any site was sufficient to mount a maximum inflammatory response. As LPS-infusion via the portal route likely resulted in a much larger total hepatic LPS load as compared to the systemic route of administration, it might be concluded that the stimulation of extrahepatic TNF-alpha generating cells resulted in comparable TNF-alpha kinetics and subsequent sequences of clinical signs.

In addition to these LPS-effects, we found an impact of chronic enteral DON-exposure. Sole DON-feeding induced a significant increase in total leukocyte counts close to the upper physiological range. This impact of DON is also reported after a 28 day exposure of oral low DON dosage (0.75–3 mg/kg) in young swine [24] and. after an i.v. DON-injection (1 mg/kg BW) in miniature pigs [25]. Their data showed a significant increase of leukocytes and neutrophils due to DON whereas other studies in growing pigs exposed to an oral low DON dosage (0.28–3 mg/kg feed) for 32 days [26,27,28] or to a 1 h DON-infusion (0.1 mg/kg/BW) [19] found no impact of DON on white blood cell counts. These DON-effects on the leukogram have been ascribed to co-contaminations with other mycotoxins and to changes in feeding behavior and thus an altered nutritional status by various authors [26,29]. In order to prevent changes in feeding behavior, pigs in our study were fed restrictively and therefore the detected impact on leukocyte counts can be attributed to the immunological response of the organism to DON.

Moreover, it is reported that DON shows interactive effects with a further immune stimulus, such as LPS, resulting in a modified APR. Although our data could not confirm this interaction on TNF-alpha, previous studies have reported on a significant attenuation of the TNF-alpha peak in pigs infused with the combination of DON (0.1 mg/kg BW) and LPS (7.5 µg/kg BW) compared to both toxins alone [15]. However, we found significantly interactive effects between DON and LPS on body core temperature and clinical symptoms but those seemed to be dependent on the site of infusion. Similar interactive effects of both toxins, irrespective of infusion site, were detected in different studies in mice treated with a single oral DON dose (25 mg/kg BW dissolved in 0.25 mL) and an intraperitoneal LPS-injection (0.5 mg/kg/BW dissolved in 0.25 mL) [30,31]. The co-exposure of LPS and the mycotoxin showed, amongst other things, an induction of apoptosis of immune cells in liver, spleen, thymus, and Peyer´s patches with a synergistic impact of both toxins on thymus and spleen [30]. Furthermore, other porcine studies showed an attenuation in hepatic histopathology when pigs were fed a DON-contaminated diet (3.1 mg/kg feed, 37 d) and received a subsequent LPS-infusion (7.5 µg/kg BW) [32]. In simultaneously DON and LPS infused pigs fed with a control diet both toxins strongly interacted with each other and elevated bilirubin levels significantly in contrast to the sole toxin administration [32]. This contrast was also verified in our experimental pigs, demonstrating a dramatic impact on development of lactic acidosis in DON-fed pigs receiving peripheral LPS [33]. Other comparable interactions for clinic and hematology are scarcely reported in pigs so far and those few reports show no significant impact of DON combined with LPS on clinical symptoms, white blood cell counts, and cytokine expression in pigs [15,34].

Furthermore, only few experimental data elucidating the liver’s involvement in this context have been published yet. Our data showed various alterations caused by infusion-site of LPS and its combination with diet. CON-fed pigs, after LPS-portal infusion, returned even more rapidly to physiological clinical levels than their post-hepatic infused counterparts. This is probably explained by the fact that a portal-infused (pre-hepatic) immune stimulus such as LPS and substances absorbed from the GIT such as DON have to pass the liver (first-pass) before reaching systemic blood circulation. Thus, those pathogenic agents could proportionally be removed by the liver to reduce the pressure for the rest of the organism, while a jugular-infused (post-hepatic) stimulus spreads through the whole blood system before passing the liver (second-pass). The latter might also explain that in our study a chronic DON exposure shortened the response time of leukocytes and accelerated the occurrence of associated clinical signs at 15 min when pigs were post-hepatically LPS-infused. Additionally, these pigs showed a significantly lower elevation of body core temperature compared to their CON-fed counterpart. This interaction between DON and LPS with respect to body core temperature also tended to be seen in portal-infused groups. To the best of our knowledge, this is the first time that such an interaction of DON-feeding and peripheral LPS-stimulus on body core temperature is reported in pigs. However, a previous study in mice could demonstrate an impact of DON *per os* on body temperature and central inflammation [35]. Therein wild-type mice, receiving an oral bolus of DON at different concentrations, exhibited a dose-dependent, transient decrease in body temperature, lasting at least 6 h. At the level of the central nervous system, mice showed, *inter alia*, significantly upregulated TNF-alpha and COX-2 transcripts in hypothalamus and dorsal vagal complex. Centrally upregulated TNF-alpha is proposed to act as an endogenous cryogen, lowering the body temperature in response to DON that is indeed observed in these mice. Consequently, we hypothesize that our DON-fed pigs experienced a similar central nervous effect, thus effectively resulting in the observed lower elevation in body temperature in response to a subsequent LPS-treatment compared to their control-fed counterparts.

Whether this DON impact on body temperature can be appraised as positive or negative for the pig’s organism remains to be elucidated. Such an increase in body temperature can positively support the inflammatory response in counteracting live pathogens, but is also considered as detrimental for the body’s tissue when passing an upper threshold. Therefore, a successful elevation in body temperature should be regulated on the lowest possible level. One could argue that the organism is more capable in successfully counteracting or even eliminating LPS-mediated effects when being exposed to prior DON-feeding.

In conclusion, the present study indicated that chronic oral DON-exposure modulates the metabolizing and detoxifying properties of the porcine liver against acute LPS stimulus. This confirms our hypothesis that the liver differently mediates the systemic inflammatory response, especially in leukocytes, body core temperature, and clinical signs.

## 4. Experimental Section

Experiment and procedures were conducted according to the European Community regulations concerning the protection of experimental animals and the guidelines of the German Animal Welfare Act and were approved by the ethical committee of the Lower Saxony State Office for Consumer Protection and Food Safety (file number 33.4-42502-04-13/1274).

### 4.1. Experimental Design

**Figure 6:**
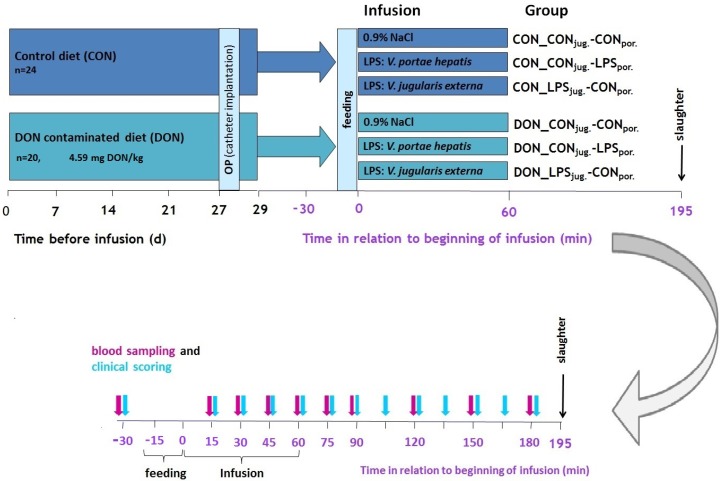
The feeding trial took place over a period of four weeks and the general experimental setup, including experimental groups is depicted in the upper panel. On day 27, pigs were surgically equipped with arterial and venous catheters followed by a recovery day. After the morning feeding (6:45–7:00) on day 29, animals were exposed to acute intravenous treatments for 1h thereby creating six experimental groups in total. (lower panel) Over a period of 210 min, starting 30 min before infusion until 180 min *p.i.* blood samples were taken and clinical signs were observed.

To investigate the effects of dietary DON combined with an intravenous LPS stimulus on acute phase reaction in pigs, a total of six experimental groups were tested (Figure 6).

### 4.2. Animals and Diets

The study was accomplished using a total of 44 barrows (German Landrace, Mariensee, Germany) with an initial mean body weight (BW) of 25.8 ± 3.7 kg. Animals were divided in two feeding-groups: one group was chronically exposed to a diet incorporating maize naturally contaminated with DON and the other group received the same diet with non-contaminated maize (Table 2). DON was measured in both compound diets using an HPLC-method as reported earlier [36] and values are provided in Table 2. During the feeding trial pigs were kept separately in floor pens and fed restrictively twice daily in equal quantity (2 × 700 g/animal and day). Thus, each pig received 6.43 mg DON per day. Animals were accustomed to balance cages and fasted the night before surgery.

**Table 2:** Composition of experimental diets (based on dry matter content of 88%).

Ingredients	CON Diet	DON Diet
	g/kg ADM	g/kg ADM
barley	533	533
maize (non contaminated)	150	75
maize (contaminated)	-	75
soybean meal	200	200
rapeseed	50	50
soybean oil	20	20
Premix ^1^	30	30
Lysine-HCl	4	4
l-Threonine	1.2	1.2
DL-Methionine	1.5	1.5
HCl-insoluble ash ^2^	10	10
**Analyzed Composition**		
crude protein	196.9	194.8
crude fat	47.5	46.5
crude ash	69.7	69.5
crude fiber	51.3	49.3
Deoxynivalenol mg/kg	0.12	4.59

^1^ Provided per kilogram of diet: Ca 0.75 g, P 0.18g, Na 0.17 g, Mg 0.03 g, Fe 120 mg, Cu 15 mg, Mn 80.1 mg, Zn 100.2 mg, I 2.01 mg, Se 0.41 mg, Co 0.25 mg, bas. Co-II-carb-monohydrat 0.25 mg, vitamin A 12,000 I.U., vitamin D3 1200 I.U., vitamin E 36 mg, vitamin B_1_ 1.13 mg, vitamin B_2_ 3 mg, vitamin B_6_ 3 mg, vitamin B_12_ 22.5 µg, vitamin K_3_ 1.58 mg, nicotinic acid 15 mg, pantothenic acid 10.13 mg, choline chloride 150 mg; ^2^ >97% SiO_2_ (Sipernat ^®^ 22S, Evonic industries, Hanau-Wolfgang, Germany).

### 4.3. Surgery

Following an initiate intramuscular application of tiletamin-zolazepam (4.4 mg/kg BW, Zoletil ^®^ 100 mg/mL, Virbac AG, Glattbrugg, Switzerland), atropine (0.04 mg/kg BW, Atropinum sulfuricum 0.5 mg Eifelfango ^®^, Eifelfango, Bad Neuenahr-Ahrweiler, Germany) and carprofen (4 mg/kg BW, Rimadyl ^®^ 50 mg/mL, Pfizer, Münster, Germany) surgery took place under sterile conditions and general inhalation anasthesia maintained with isofluran (Isofluran CP, CP-Pharma, Burgdorf, Germany).

Pigs were equipped with five catheters (Silastic ^®^ Medical Grade Tubing, 1.57 mm ID × 3.18 mm OD, Dow Corning, Midland, MI, USA) covering pre- and post-hepatic area for contemporaneous blood sampling and infusions. Beginning with a median laparotomy first catheter was put in via an access by a jejunal vein through the cranial mesenteric vein into the *Vena portae hepatis* for sampling from the portal drained viscera (PDV). The second catheter goes via an access by a vein of the great curvature to the *Vena splenica* for applications. After being fixed catheters were exteriorized subcutaneously to the left flank and attached with catheter clamps with fastener (Arrow ^®^, Arrow International Inc., Reading, PD, USA). Additionally, a temperature logger (Thermochron i-Button, Maxim integrated™, San Jose, CA, USA) was fixed in the abdominal cavity and subsequently abdominal incision was closed continuously in two layers. Thereafter, the left jugular grove was opened to insert a catheter each into the *Vena jugularis externa* for applications, *Vena jugularis interna* for sampling from the post-hepatic area and *Arteria carotis communis* for sampling as well. After being fastened, they were tunnelled subcutaneously to the neck and the incision in the jugular grove was closed with skin suture. Catheters of the portal vein and the carotid artery were fixated in the vessel using pursestring suture (Premilene ^®^ 5/0, B.Braun Melsungen AG, Melsungen, Germany) whereas remaining catheters were inserted as previously described in detail (Goyarts and Dänicke, 2006 [10]) and each catheter was provided with three-way-valves for blood sampling and infusions. Heparinized 0.9% NaCl was used for flushing catheters to maintain patency.

### 4.4. Infusions, Clinic, and Sampling

All infusions were performed using an infusion-pump (IPC-N-4, ISMATEC Laboratoriumstechnik GmbH, Wertheim, Germany) and infusion-tubes with 2.06 mm inner diameter (PharMed ^®^ Ismaprene, ISMATEC Laboratoriumstechnik GmbH) at a rate of 32 mL/h per animal and infusion catheter. Infusion was administered for 60 min simultaneously into both, *V. jugularis externa* (post-hepatic) and *V. splenica* (pre-hepatic), in each experimental animal. Control groups were infused with physiological saline in both catheters (CON-CON_jug._-CON_por._; DON-CON_jug._-CON_por._), while LPS (7.5 µg/kg BW dissolved in 0.9% NaCl, *Escherichia coli* O111:B4, Product number L2630, Sigma-Aldrich, St. Louis, MO, USA) was administered either into jugular (CON-LPS_jug._-CON_por._; DON-LPS_jug._-CON_por._) or portal region (CON-CON_jug._-LPS_por._; DON-CON_jug._-LPS_por._) and the second catheter was simultaneously infused with physiological saline.

Over a period from −30 min before to 180 min after starting the infusion, different clinical signs were scored (Table 3) and respiratory rate was counted at −30, 15, 30, 45, 60, 75, 90, 105, 120, 135, 150, 165, and 180 min. In addition, body core temperature was measured by an i-Button every 5 min. Furthermore serial blood samples were collected for leukocyte count, differential white blood cell count (EDTA Monovette^®^, Sarstedt AG & Co., Sarstedt, Germany) and TNF-alpha (Li-Heparin Monovette ^®^, Sarstedt AG & Co.) at −30, 15, 30, 45, 60, 75, 90, 120, 150, and 180 min. Samples for cytokine analyses were immediately centrifuged and plasma samples were stored at −20 °C for subsequent investigations.

**Table 3:** Clinical score: Cumulative score calculated from all scores of each symptom over the whole observation period (10 symptoms × 13 times, maximum score 351) or of each symptom for every point in time (10 symptoms per time, maximum score 27).

Clinical Symptom		Scores
**respiratory rate**	10–20/min	0
	21–40/min	1
	41–60/min	2
	61–80/min	3
	>80/min	4
**respiratory difficulties**	none	0
	low labored breathing	1
	medium labored breathing	2
	severe labored breathing	3
	open-mouth breathing	4
**tremor**	none	0
	low shivering	1
	medium shivering	2
	severe shivering	3
	spasms	4
**hyperaemic conjunctivae**	physiological	0
	(rose) red	1
	red	2
**injected episcleral vessels**	none	0
	slightly injected	1
	medium injected	2
	highly injected	3
**cyanosis**	none	0
	low cyanosis	1
	medium cyanosis	2
	severe cyanosis	3
**vomiting, retching**	none	0
	smacking, foam-forming, retching	1
	vomiting of slime	2
	vomiting of feed/digesta	3
	vomiting of slime and feed/digesta	4
**dermographism**	none	0
	skin coloring pattern present	1
**teeth gnashing**	none	0
	teeth gnashing present	1
**nystagmus**	none	0
	nystagmus present	1

### 4.5. Measurements and Analyses

#### 4.5.1. Clinic

At each time clinical symptoms were examined and a score system implemented to facilitate objective comparisons (Table 3). Scoring was performed by one person only, who was unaware of animal´s treatment at time of clinical observations. These 10 scored clinical signs were summarized as cumulative clinical score as a measure of clinical severity, on the one hand comprising all 13 times and each clinical symptoms with a total score of maximal 351, and on the other hand comprising all symptoms for each point in time seperatly with a total score of maximal 27 per time.

#### 4.5.2. White Blood Cell Counts

Serial whole blood samples were anaylyzedimmediately after sampling by an automatic hematology analyzer (Celltac MEK 6400, Nihon Kohden Europe GmbH, Rosbach, Germany) for total leukocyte counts.

Additionally, one drop of blood was placed on a glass slide and two blood smears were prepared of each blood sample in order to differentiate the leukocyte types in duplicates. After air-drying blood smears were stained according to pappenheim: Slides were places into may-grÜnwald solution for fixation and staining for 3 min and subsequently rinsed in distilled water (pH 7.2) to remove staining solution. After that, slides were put into giemsa solution for counterstaining (15 min) and again rinsed in distilled water (pH 7.2). Dried samples were analyzed using brightfield microscopy (Nikon Eclipse E200, Nikon GmbH, Tokyo, Japan) for morphological differentiation of 100 leukocytes per slide.

#### 4.5.3. TNF-Alpha

TNF-alpha was determined at defined times (−30, 30, 60, 90, 120, 180 min) using a quantitative ELISA kit (Quantikine^®^ ELISA Porcine TNF-α Immunoassay, Cat. No. PTA00, R & D System Inc., Minneapolis, MN, USA) according to the manufacturers manual with a level of detection of 2.8–5 pg/mL. Samples were measured photometrically on a Tecan (Tecan^®^ infinite M200, Tecan Trading AG, Männedorf, Switzerland) at 450, 540, and 570nm.

### 4.6. Statistics

All statistics were performed by using SAS software (Enterprise Guide, version 6.1, SAS Institute, Cary, NC, USA). Generally, the procedure “MIXED” was used with group (six experimental groups), sampling site (three locations: *A. carotis comunis*, *V. jugularis interna*, *V. portae hepatis*), time (10 points in time for blood samples, 13 points in time for clinical scores) and their interactions included as fixed factors with a compound symmetry covariance structure for leukocyte counts and TNF-alpha measurements. Body temperature and clinical signs did not take the sampling site as a fixed factor into consideration and used an autoregressive covariance structure cumulative score.

Effects were regarded to be significant (adjusted Tukey post-hoc test) at likelihood lower or equal to 0.05 while a tendency or trend was assumed for probabilities lower than 0.1 and higher than 0.05.

## 5. Conclusions

Altogether we were able to show both an effect of sole chronic DON-feeding and an infusion-site effect of the subsequent LPS-stimulus. Based on our data and data from literature, we suggest that chronic DON exposure, in combination with a subsequent immune stimulus, on the one hand overstrains the functional capacity of the liver as indicated by the earlier and stronger leukopenia and on the other hand accelerates the innate and adaptive immune response as evidenced by the more uniform return of clinical symptoms to physiological levels combined with lower levels of hyperthermia.
